# The effect of COVID‐19 and COVID‐19 vaccination on serum anti‐Mullerian hormone: A systematic review and meta‐analysis

**DOI:** 10.1002/iid3.1136

**Published:** 2024-01-10

**Authors:** Marjan Ghaemi, Sedigheh Hantoushzadeh, Arman Shafiee, Omid Kohandel Gargari, Hanieh Fathi, Nasim Eshraghi, Jafar Razavi, Gholam Reza Habibi, Kyana Jafarabady

**Affiliations:** ^1^ Vali‐E‐Asr Reproductive Health Research Center, Family Health Research Institute Tehran University of Medical Sciences Tehran Iran; ^2^ Student Research Committee, School of Medicine Alborz University of Medical Sciences Karaj Iran; ^3^ Department of Psychiatry and Mental Health Alborz University of Medical Sciences Karaj Iran

**Keywords:** anti‐Mullerian hormone, COVID‐19, fertility, vaccine

## Abstract

**Objective:**

The current study aims to evaluate the impact of COVID‐19 infection and vaccination on ovarian reserve by detecting the anti‐Mullerian hormone (AMH) level.

**Method:**

PubMed, Embase, Web of Science, and Scopus has been searched for studies assessing the effect of COVID‐19 infection and/or vaccination on AMH levels up to February 27, 2023. Based on PRISMA 2020 statement criteria, a systematic review and meta‐analysis of included studies were performed. The studies' quality was assessed by the National Institute of Health (NIH) quality assessment tool. The standardized mean difference (MD) of the AMH level was used and the quantitative values of each study were pooled separately by using a random effect model.

**Results:**

Out of 246 studies screened, 18 were included in the systematic review and 14 in the meta‐analysis. Included studies were published between 2021 and 2022 and were conducted in different countries, including the USA (*n* = 3), China (*n* = 2), Russia (*n* = 2), Turkey (*n* = 5), Israel (*n* = 3), Czech (*n* = 2), and Spain (*n* = 1). Eight studies investigated the effect of SARS‐CoV‐2 infection on AMH levels, and ten studies investigated the possible effect of COVID‐19 vaccination on AMH levels. The pooled analysis showed a statistically significant decrease in AMH levels after COVID‐19 infection (SMD: −0.24; 95% CI: −0.36 to −0.11; I2 = 0%; *p* = .0003). Vaccination analysis showed a nonstatistically significant change in AMH levels after COVID‐19 vaccination (SMD: −0.11; 95% CI: −0.25 to 0.04; I2 = 35%; *p* = .14).

**Conclusion:**

COVID‐19 infection can result in ovarian reserve injury by reducing the AMH level but getting vaccinated against COVID‐19 has no impact on the AMH level.

## Highlights

- Anti‐Mullerian hormone (AMH) level is found to be impacted by COVID‐19 infection.
- AMH level is not affected by Vaccination against COVID‐19.
- Vaccination can prevent ovarian reserve injuries by decreasing the severity of COVID‐19 infection and further complications in pregnancy.

## Graphical abstract

COVID‐19 infection can result in ovarian reserve injury by reducing the anti‐Mullerian hormone (AMH) level but getting vaccinated against COVID‐19 has no impact on the AMH level.

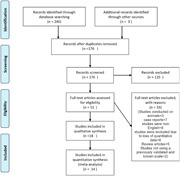

## 1 INTRODUCTION

Since early December 2019, when the COVID‐19 pandemic began, billions of people worldwide have been impacted in various ways and the pandemic has presented immense challenges for public health.^1^ The Severe Acute Respiratory Syndrome Coronavirus‐2 (SARS‐CoV‐2) is primarily transmitted through respiratory droplets and physical contact.^2^ COVID‐19 has been found to affect not only the respiratory system, but also a range of other organs, including the cardiovascular, immune, and urinary systems, leading to a variety of disorders such as coagulopathy, disseminated intravascular coagulation, sepsis, and acute kidney injury.^3^, ^4^ Initially, the primary measures to combat COVID‐19 were self‐distancing, mask‐wearing, and isolating infected patients, however, the advent of the first vaccines in December 2020 marked a significant turning point in the fight against the pandemic, as they have been shown to reduce the escalation of both the number of deaths and the severity of the disease.^5^, ^6^, ^7^, ^8^ Since then, over 11 billion vaccine doses have been administered worldwide.^9^, ^10^ Although the vaccine has played a vital role in the pandemic,, it is reported that vaccination can lead to short‐term menstrual distributions.^11^ Coronavirus particles contain a spike protein (S) on their outer surface which can bind to the human cell surface receptor of angiotensin‐converting enzyme 2 (ACE2) strongly which can trigger the immune system.^12^ Since the ACE2 receptors are highly expressed in uterine, placental, and ovarian cells, there would be possible if COVID‐19 infection can affect fertility in women.^13^ Although the effect of vaccination against COVID on fertility is still uncertain, but many studies revealed that vaccination would not affect ovarian reserve.^14^, ^15^, ^16^ It is reported that vaccination can lead to short‐term menstrual alterations.^11^, ^17^, ^18^, ^19^

Anti‐Müllerian hormone (AMH) is a glycoprotein produced by the ovarian preantral and small antral follicles which are known as a predictor of ovarian reserve estimation.^20^, ^21^ AMH is the only reproductive hormone that is not influenced by different states of the menstrual cycle.^21^ And accounts for the prediction of ovarian response to ovarian stimulation.^22^

There are studies suggesting that COVID‐19 vaccination does not have an effect on male or female fertility,^5^ although it is questioned whether vaccines could have a subclinical effect on female fertility factors such as AMH.

Due to the immense number of women who have gotten infected by COVID‐19 or get vaccinated against it, the present systematic review and meta‐analysis is conducted to evaluate the effect of COVID‐19 infection and vaccination on women's fertility by their influence on AMH.

## 2 METHOD

### 2.1 Search strategy

The present systematic review and meta‐analysis have been conducted in accordance with the Cochrane Handbook.^23^ We have used The Preferred Reporting Items for Systematic Reviews and Meta‐Analyses (PRISMA) for the current study.^24^ Our study protocol is registered at PROSPERO under the number CRD42023420512. PubMed, Embase, Web of Science, and Scopus have been searched by our reviewers, using the following search string: “Anti‐Mullerian Hormone” [mh] OR Anti Mullerian Hormone [tiab] or [tiab] or Mullerian‐Inhibiting Factor [tiab] or [tiab] or Mullerian Inhibiting Factor [tiab] or [tiab] or Anti‐Mullerian Factor [tiab] or [tiab] or Anti Mullerian Factor [tiab] or [tiab] or Mullerian‐Inhibitory Substance [tiab] or [tiab] or Mullerian Inhibitory Substance [tiab] or [tiab] or Mullerian Inhibiting Hormone [tiab] or [tiab] or Mullerian Inhibiting Substance [tiab] or [tiab] OR Mullerian Regression Factor [tiab] or [tiab] or Mullerian‐Inhibiting Hormone [tiab] or [tiab] or Anti‐Muellerian Hormone [tiab] or [tiab] or Anti Muellerian Hormone [tiab] or [tiab] or [tiab] or Antimullerian Hormone [tiab] or [tiab] or AMH [tiab] and 2‐ (“COVID‐19” [Mesh]) or (“SARS‐CoV‐2” [Mesh]) or (COVID‐19 [Title/Abstract]) OR (Coronavirus[Title/Abstract]) or (vaccin*[Title/Abstract]) or (SARS‐Cov‐2[Title/Abstract])). Up to 27 February all the publications have been retrieved. The detailed search strategy used for each database is provided in supplementary file. For further screening, all the results have been exported to the EndNote X9 program.

### 2.2 Study selection and data extraction

The inclusion criteria were as follows^1^: patient population: women of reproductive age after getting infected by COVID‐19 or getting vaccinated against it with at least one dose^2^; Intervention: Vaccination with COVID‐19 vaccines or getting infected by COVID‐19^3^; Comparison: women of reproductive age not vaccinated against COVID‐19 or prior vaccination against COVID‐19 or women of reproductive age who have not been infected by COVID‐19^4^; Outcome: impact on the Anti‐Mullerian hormone (AMH) level^5^; Setting/Time: All and^6^ study design: randomized controlled trial, retrospective studies, and prospective studies.

Exclusion criteria were as follows: studies conducted on animals or minors and all study design other than the ones which were mentioned before including case reports, and case series.

Articles based on their title, abstract, and then full text has been screened by two reviewers in the Endnote, independently. All disagreements were resolved via discussion with a third reviewer to reach an agreement. Data were extracted via texts, tables, graphs, supplementary materials, and figures and were imported into an excel spreadsheet. Two reviewers have extracted characteristics data including Author, country, and year of publication, number of patients in each study, study type, and outcomes.

### 2.3 Quality assessment

Two reviewers assessed the quality of the included studies using the National Heart, Lung, and Blood Institute (NHLBI)/National Institutes of Health (NIH) Quality Assessment tool. For the prepost studies, they used a 12‐item assessment, while for the other studies, a 14‐item assessment was employed.^25^ Studies were classified into three groups (good, fair, and poor) based on the scoring. Disagreements were resolved by discussion with the third reviewer.

### 2.4 Outcome measure

Our outcome was assessing female fertility by measuring the effect of COVID‐19 and COVID‐19 vaccination on AMH level.

### 2.5 Data synthesis and analysis

The standardized mean difference (MD) of the AMH level was used and the quantitative values of each study were pooled separately by using a random effect model. Excel calculator was used to estimate the MD if it was not reported. For assessing heterogeneity, Cochran's Q statistic (Q‐test) and the I2 were used. I2 value > 75% indicated a high amount of heterogeneity. visual inspection of the funnel plot was used to assess publication bias and Egger's test with a significance level of 0.05 was used to evaluate the publication bias. Associations where the *p*‐value < .05 were considered significant. RevMan 5.4 and R‐4.1.3 software and the Meta package (R Core Team, Vienna, Austria; available at https://www.R-project.org/, accessed on 6, January 2022) were used for analysis.

## 3 RESULTS

### 3.1 Literature search

**Figure 1:**
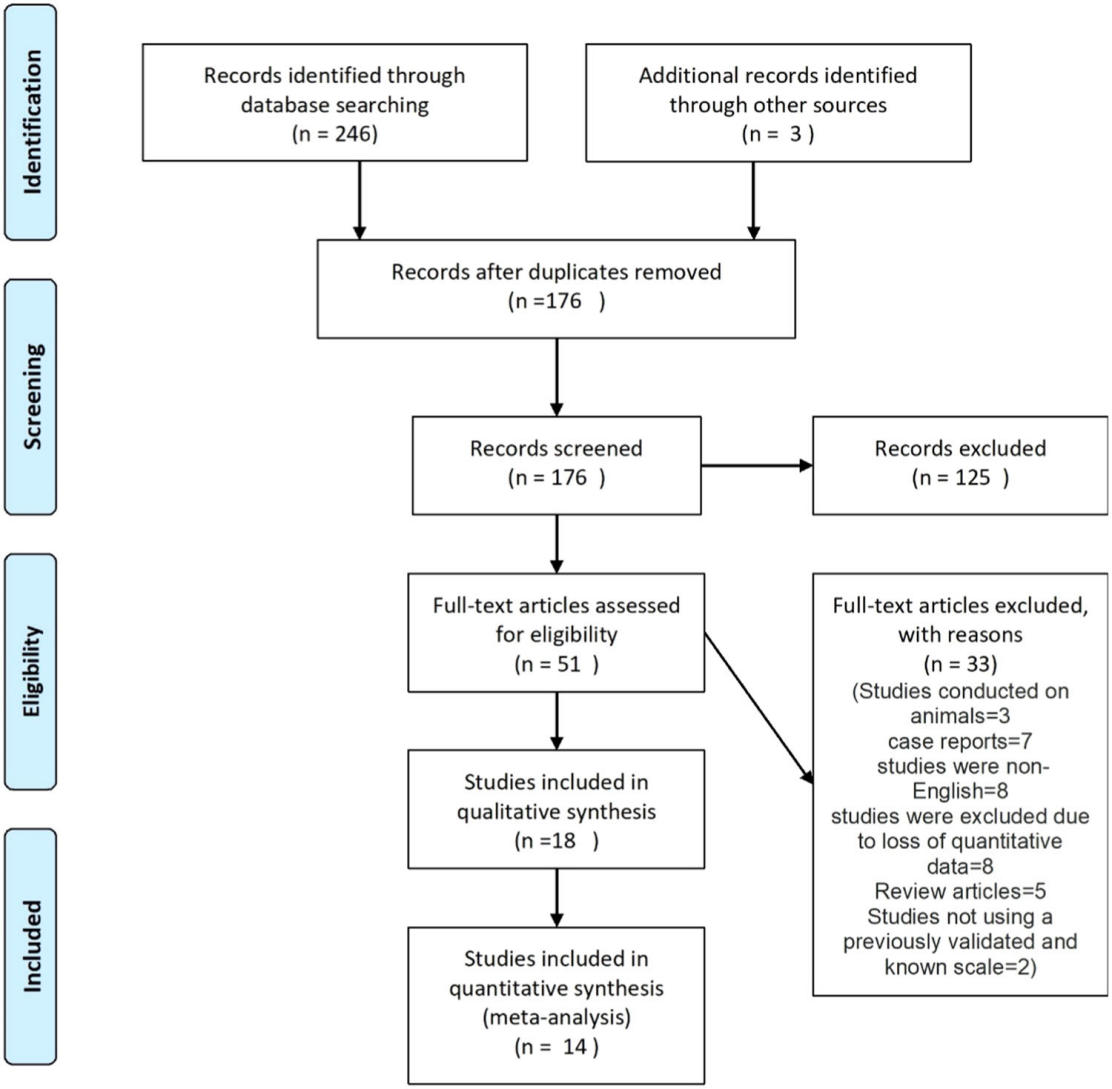
PRISMA flow diagram.

The initial database search yielded a total of 264 studies. After removing duplicates and screening based on title and abstract, 173 studies were identified for full‐text review. After applying inclusion and exclusion criteria, a total of 15 studies were included in the final systematic review. The study selection process is presented in the PRISMA flow diagram (Figure 1).

### 3.2 Baseline characteristics

The 18 included studies were published between 2021 and 2023 and were conducted in different countries, including the USA (*n* = 3), China (*n* = 2), Russia (*n* = 2), Turkey (*n* = 5), Israel (*n* = 3), Czech (*n* = 2) and Spain (*n* = 1). Eight studies investigated the effect of SARS‐CoV‐2 infection on AMH levels.^26^, ^27^, ^28^, ^29^, ^30^, ^31^, ^32^, ^33^, ^34^, ^35^, ^36^ Ten studies investigated the possible effect of COVID‐19 vaccination on AMH levels.^16^, ^34^, ^35^, ^36^, ^37^, ^38^, ^39^, ^40^, ^41^, ^42^ Six studies used m‐RNA vaccines, one used the Russian GAM‐COVID vaccine, two studies used CoronaVacc, and two did not report the type of vaccines used. All studies were observational, including prospective and retrospective studies. The total number of participants included in the COVID‐19 analysis was 618, and the total number of participants included in the vaccination analysis was 1681.

### 3.3 Quality assessment

The quality assessment using the National Heart, Lung, and Blood Institute (NHLBI) showed that no study was rated as poor quality, with specific detail about each study assessment rate in Table S3.

### 3.4 COVID‐19 analysis

**Figure 2:**
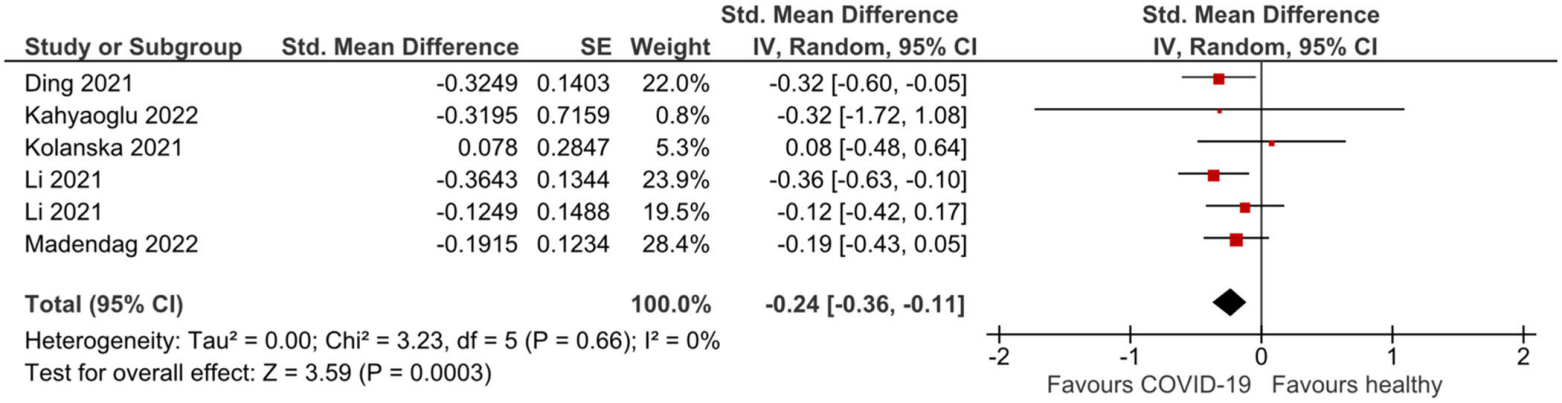
Results of a COVID‐19 analysis.

The detailed characteristics of the included studies in COVID‐19 analysis are presented in Table 1. The main outcome of this analysis was the effect of COVID‐19 infection on AMH levels in women of reproductive age. The pooled analysis showed a statistically significant decrease in AMH levels after COVID‐19 infection (SMD: −0.24; 95% CI: −0.36 to −0.11; I2 = 0%; *p* = .0003) (Figure 2). Subgroup analysis based on study design did not show a statistically significant between‐group difference (*p* = .6910). The funnel plot showed no evidence of publication bias (Figures S1 and S2), and Egger's test was not applicable because fewer than 10 studies were included in our analysis. Furthermore, the sensitivity analysis results did not alter the statistically significant association (Figure S3).

**Table 1:** Characteristics of studies investigating the effect of COVID‐19 on anti‐müllerian hormone (AMH) levels.

Author	Year	Type of study	Design	Country	COVID‐19 severity	Total patients	Age	Main findings
Coyne	2022	Observational	Before and after	USA	NR	22	18–41	1‐no significant difference was reported in AMH level at baseline and 3‐ or 6‐months post COVID‐19 2‐ *p* = .08
Cruz	2021	Observational	Before and after	Spain	NR	46 (16 women were diagnosed as having low ovarian reserve (AMH < 1 ng/mL) and 30 were classified as having normal ovarian reserve (AMH ≥ 1 ng/mL)	34.7 years in normal ovarian reserve and 38.6 years in low ovarian reserved	1‐no significant changes in AMH level before and after COVID infection 2‐ in women with normal ovarian reserve, AMH level decreases more than in women with low ovarian reserve
Ding	2021	Observational	Case and control	China	Both mild and severe patients	78 (17 were diagnosed as severe and 39 were basal group)	43.50 (36.75–47.00)	1‐COVID‐19 patients have decreased AMH *p* = .001 2‐covid infection cause ovarian injuries
Kahyaoglu	2022	Retrospective	Before and after	Turkey	Mild	4	21–40	1‐ with *p* = .54 there were no significant changes before and after COVID infection
Kolanska	2022	Prospective observational study	Case and control	Czech	Mild	118	20–43	1‐COVID‐19 infection can not affect the AMH level significantly
Li	2021	retrospective, cross‐sectional	Case and control	China	Both mild and severe patients	177	18 and 45 years	1‐ AMH level did not change significantly
Madendag	2022	Observational	Before and after	Turkey	Both mild and severe patients	132	28 (23–34)	1‐no differences of AMH between pre and postillness (*p* = .097)
Ermakova	2022	prospective observational study	Before and after	Russia	Mild and moderate	41	late reproductive age (LRA) ( > 35 years)	1‐ in late reproductive age COVID infection can decrease the ovarian reserve.

### 3.5 Vaccination analysis

**Figure 3:**
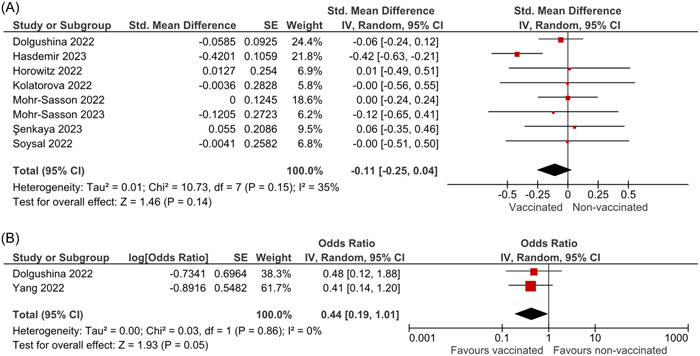
Results of a vaccination analysis.

The detailed characteristics of the included studies in Vaccination analysis are presented in Table 2. The main outcome of this analysis was the effect of COVID‐19 vaccination on AMH levels in women of reproductive age. The pooled analysis showed a nonstatistically significant change in AMH levels after COVID‐19 vaccination (SMD: ‐0.11; 95% CI: −0.25 to 0.04; I2 = 35%; *p* = .14) (Figure 3A). Subgroup analysis based on study design did not show a statistically significant between‐group difference (*p* = .69). The funnel plot showed no evidence of publication bias (Figures S4 and S5), and Egger's test was not applicable because fewer than 10 studies were included in our analysis. Furthermore, the sensitivity analysis results did not alter the nonstatistically significant association (Figure S6). In addition to the above analysis, we performed a binary meta‐analysis on the odds of lower than predefined cut‐off AMH after vaccination, our results showed a nonstatistically significant trend regarding the protective effect of vaccination on AMH (OR: 0.44; 95% CI: 0.19 to 1.01; I2 = 0%; *p* = .05) (Figure 3B).

**Table 2:** Characteristics of studies investigating the effect of COVID‐19 vaccination on anti‐müllerian hormone (AMH) levels.

Author	Year	Type of Study	Design	Country	Total patients	Age	Vaccine	Main findings
Dolgushina, N. V.	2022	Prospective interventional study	Before and after	Russia	220	33 (26–39)	Russian Gam‐COVID vaccine	1‐Vaccine has no adverse effect on ovarian reserve in women
								2‐*p*‐value for AMH before and after the vaccine is .794
Horowitz, E.	2022	Prospective observational study	Before and after	Israel	31	35.5 ± 4.7	mRNA COVID‐19 vaccine	1‐The median AMH concentrations before and after COVID‐19 vaccine did not change significantly (*p* = .96).
								2‐No correlation was found between the participant's anti‐COVID‐19 antibody titer and the change in AMH concentration.
Kolatorova, L.	2022	Observational study	Before and after	Czech Republic	25	30 ± 6.8	Pfizer/BioNTech or Moderna	1‐With *p* = .689 there are no significant changes before and after the vaccine
Mohr‐Sasson, A.	2022	Prospective study	Before and after	Israel	129	29.3 ± 5.2	mRNA COVID‐19 vaccine	1‐Mean AMH levels were 5.3 (§SD 4.29) mg/L and 5.3 (§SD 4.50) mg/l before vaccination and after 3 months, respectively (*p* = .11)
								2‐ There was no significant difference before and after the vaccine
Palmerola, K. L.	2022	Retrospective analysis	Case and control	USA	106	NA	NA	1‐There was no significant difference between the two groups for the AMH level (*p* > .05).
Soysal, Ç	2022	Prospective cross‐sectional study	Before and after/case and control	Turkey	60	case=27.30 ± 1.66/control=27.40 ± 1.69	mRNA COVID‐19 vaccine	1‐There was no significant difference before and after vaccination. *p* > .05
Yang, L.	2022	Retrospective cohort study	Before and after	USA	771	NA	NA	1‐There was no change in AMH (OR 0.41 95% CI 0.14‐1.14) The mean AMH before vaccination was 4.2 and post was 5.2.
Hasdemir,P	2023	Prospective cohort study	Before and after	Turkey	258	35.0 (12.0)	CoronaVac+BioNTech	1‐A significant decrease in AMH level after 6 months was reported. (*p* = .001)
								2‐AMH level was significantly increased after 9 months in comparison with 6 months postvaccination (*p* < .001)
								3‐vaccination can cause a transient decrease in AMH level which is reversible.
Şenkaya AR	2023	Retrospective cohort study	Before and after	Turkey	46	36.4 ± 4.9	CoronaVac	1‐AMH level did not changed significantly before and after vaccination (*p* = .366)
Mohr‐Sasson, A.	2023	Prospective cohort study	Before and after	Israel	35	13.8 ± 1.3	mRNA vaccine	1‐ AMH level did not change significantly before and after vaccination (*p* = 0.07)
								2‐vaccination does not seem to have an effect on AMH level.

## 4 DISCUSSION

The present systematic review aimed to investigate the effect of SARS‐CoV‐2 infection and COVID‐19 vaccination on anti‐Mullerian hormone (AMH) levels in women of reproductive age. Our search yielded 15 studies that met the inclusion criteria, including eight studies that investigated the effect of SARS‐CoV‐2 infection on AMH levels and seven studies that investigated the effect of COVID‐19 vaccination on AMH levels. The results indicated a significant decrease in AMH levels following SARS‐CoV‐2 infection in women of reproductive age. On the other hand, our analysis showed a nonsignificant change in AMH levels after COVID‐19 vaccination. However, the binary meta‐analysis showed a nonsignificant trend regarding the protective effect of vaccination on AMH levels. The sensitivity analysis did not alter the significant association found, which indicates the robustness of our results. It is important to note that the studies included in our analysis used different types of COVID‐19 vaccines, with four studies using mRNA vaccines and one study using the Russian GAM‐COVID vaccine. Additionally, two studies did not report the type of vaccine used.

In a systematic review and meta‐analysis study by Zaçe et al. researchers reviewed effect of COVID‐19 vaccination (Gam‐COVID‐Vac and BNT162b2) on different outcomes such as sperm characteristics, steroid hormones levels, biochemical and clinical pregnancy rate; no significant finding was reported and authors concluded that COVID‐19 vaccinations is not associated with impaired fertility outcomes.^5^ Vaccination with mRNA vaccines also showed no change in gametes and embryo parameters.^43^ Hasdemir et al. study was the only one reporting a significant decrease in level of AMH among vaccinated patients, they compared AMH level among healthcare workers pre and post vaccination. They measured AMH levels several time post vaccinations and AMH levels were only measured once before vaccination. The fact that COVID‐19 was not an exclusion criterion in their study, plus participants being health care workers with high exposer to SARS‐CoV‐2 virus, could affect the findings of this study. Although it must be noted that level of AMH was above normal range and the increase was mostly transient.^44^ Overall COVID‐19 vaccination seems to be safe regarding female laboratory and clinical fertility outcomes.

COVID‐19 could affect menstrual cycles in women and cause irregular menstruation, oligomenorrhea and increase premenopausal syndrome.^45^ In a systematic review and meta‐analysis by Cai et al. effect of COVID‐19 on sex steroids was reviewed. Patients with COVID‐19 history had significantly lower testosterone to LH ratio, FSH to LH ratio and sex hormone‐binding globulin (SHBG) levels. Levels of LH was higher in COVID‐19 patients compared to control.^1^ Although both our study and Cai et al. study found a significant alternation in fertility related lab findings but there is no long term follow up studies assessing fertility and fecundity of patients with a history of COVID‐19.

To discuss the possible underlying pathogenesis, we know that SARS‐CoV2 uses Angiotensin converting enzyme (ACE) receptor to infect the host cells.^46^ On the other hand, there is evidence regarding presence of this receptor in ovaries,^47^ so the effect of COVID‐19 on AMH level, which is reported in this study, could be due to direct involvement of ovaries. This statement is consistent with findings of a study by Naigaonkar et al. that reported a lower rate of ovarian involvement among COVID‐19 women with less “coronavirus‐associated receptors and factors”.^48^ Transmembrane serine protease 2 (TMPRSS2) is the other entry molecule of SARS‐CoV‐2 and co‐expression of TMPRSS2 and ACE is also observed in ovary cells.^49^ Another possible mechanism could be the effect of hyperinflammatory state caused by COVID‐19.^50^ We know that COVID‐19 is associated with hyperinflammatory immune response that could lead to multi organ damages including ovaries and level of AMH.^51^

Further research is required to address whether the effect of COVID‐19 and vaccination on AMH is permanent or temporary, and long term follow up studies should be conducted to assess reproductive outcomes. The available evidence is limited to suggest that there may be variations in the influence on AMH levels depending on the type of vaccine administered. Studies have particularly focused on mRNA vaccines, such as those developed by Pfizer‐BioNTech and Moderna, as well as viral vector vaccines, including those by AstraZeneca and Johnson & Johnson. Hasedemir et al. reported individuals vaccinated with CoronaVac had significantly lower serum AMH level than those vaccinated with CoronaVac+BioNTech.^34^

However, it is important to note that findings across studies are not uniform, and additional research is needed to elucidate the specific mechanisms and long‐term implications of COVID‐19 vaccination on reproductive health. Moreover, the heterogeneity in study designs, populations, and methodologies underscores the complexity of this relationship, necessitating further exploration for a comprehensive understanding of the interplay between different COVID‐19 vaccines and AMH levels. The strengths of this study include the comprehensive literature search, strict inclusion and exclusion criteria, quality assessment of included studies and low heterogeneity among included studies. However, this study has some limitations. Firstly, low number of included studies in each analysis and different types of vaccines compared together.

In conclusion, this systematic review provides evidence of a significant decrease in AMH levels after COVID‐19 infection in women of reproductive age. However, we found no significant change in AMH levels after COVID‐19 vaccination. These findings suggest that COVID‐19 infection may have a negative impact on ovarian reserve, and further research is required to assess reproductive outcomes among COVID‐19 survivors. Nevertheless, the effect of COVID‐19 vaccination on ovarian reserve needs further investigation, given the limited number of studies included in our analysis. Overall, our findings provide valuable insights into the impact of COVID‐19 on female reproductive health and may inform clinical practice and public health policies.

## AUTHOR CONTRIBUTIONS

**Marjan Ghaemi**: Conceptualization; data curation; supervision. **Sedigheh Hantoushzadeh**: Supervision. **Omid Kohandel Gargari**: Methodology. **Hanieh Fathi**: Writing—review and editing. **Jafar Razavi**: Investigation; writing—review and editing. **Gholam Reza Habibi**: Project administration; writing—review and editing. **Kyana Jafarabady**: Project administration; writing—original draft.

## CONFLICT OF INTEREST STATEMENT

The authors have no relevant affiliations or financial involvement with any organization or entity with a financial interest in or financial conflict with the subject matter or materials discussed in the manuscript.

## ETHICS STATEMENT

The authors have nothing to report.

## Supporting information

Supporting information.

## Data Availability

All data generated or analyzed during this study are included in this published article.
